# Inclusion of purified dietary fiber during gestation improved the reproductive performance of sows

**DOI:** 10.1186/s40104-020-00450-5

**Published:** 2020-05-12

**Authors:** Yong Zhuo, Bo Feng, Yuedong Xuan, Lianqiang Che, Zhengfeng Fang, Yan Lin, Shengyu Xu, Jian Li, Bing Feng, De Wu

**Affiliations:** Key Laboratory for Animal Disease-Resistant Nutrition of the Ministry of Education of China, and Animal Nutrition Institute, Sichuan Agricultural University, 211 Huimin Road, Wenjiang, Chengdu, 611130 People’s Republic of China

**Keywords:** Dietary fiber, Gut microbiota, Immunity, Reproductive performance, Sow

## Abstract

**Background:**

This study aimed to investigate the impacts of guar gum and cellulose as the source of dietary fiber during gestation on the reproductive performance of sows.

**Methods:**

A total of 210 sows (parities 3–6) were randomly allocated into six diets (*n* = 35) throughout gestation to feed graded levels of dietary fiber (DF), including a corn-soybean meal-based control diet with no wheat bran inclusion (CON, 12.5% DF), a wheat bran-rich diet (DF1, 17.4% DF), and another 4 diets (DF2, 17.7% DF; DF3, 18.1% DF; DF4, 18.4% DF; DF5, 18.8% DF) in which wheat bran were equally substituted by 1%, 2%, 3% and 4% purified FIBER MIX (guar gum and cellulose, 1:4). All sows received similar DE and other nutrients throughout gestation.

**Results:**

DF treatment during gestation resulted in normal fecal score (1 to 5 with 1 = dry and 5 = watery) in sows compared with those received the CON diet (*P* <  0.05). The number of total born piglets had a tendency to be affected by dietary treatment (*P* = 0.07), and correlation analysis revealed a linear response of total born to dietary fiber levels during gestation (*P <* 0.01). Sows received the DF2, DF3, and DF5 diets during gestation had a greater ADFI during lactation compared with those in the CON group (*P <* 0.05) without affecting the daily body weight gain of suckling piglets. Gut microbiota compositions were dramatically changed by the gestation stage and some of those were changed by DF inclusion. Fecal acetate, propionate, and butyrate of sows were markedly increased in late gestation, and butyrate contents in feces of gestating sows were significantly affected by DF levels (*P <* 0.01). Serum concentrations of pro-inflammatory TNF-α were decreased and anti-inflammatory IL-10 was increased on day 30 of gestation by DF levels (*P <* 0.05).

**Conclusions:**

In summary, increasing dietary fiber levels by guar gum and cellulose during gestation improved the reproductive performance of sows, which might be related to changes in immunity and gut microbiota of sows.

## Background

The beneficial effects of dietary fiber on the behavior and welfare of gestating sows that are restricted-fed are generally accepted and were recently reviewed by Jarrett and Ashworth [1]. Feeding high fiber diets during gestation improved litter size [2–4], piglet birth weight [3], growth rate of suckling piglets [3, 5–7], piglet viability [7, 8], and within-litter uniformity [2, 9, 10]. The inclusion of dietary fiber in most of the studies was achieved by formulating high levels of fiber-rich ingredients, e.g. sugar beet pulp and soybean hulls. However, there are many different nutrients contained in those ingredients and it’s hard to assess whether those beneficial effects were exclusively attributed to dietary fiber. Additionally, there is not any clear optimal level of dietary fiber for gestating sows [1].

The beneficial effects of dietary fiber could be attributed to different aspects, which are related to both direct effects (e.g. by gut fill) and indirect effects by the production of physiologically active by-products (e.g. short-chain fatty acids, SCFAs) following microbial fermentation in the gastrointestinal tract [1]. Gut microbiota has been shown to impact host nutritional, physiological, and immunological processes in various ways across diverse species [11, 12]. Disruption of maternal gut microbiota during gestation was noted to affect both maternal and offspring microbiota and immunity [13]. As an important energy source for gut microbiota, dietary fiber interacted directly with gut microbes to improve host metabolism and immune function via SCFAs [14, 15]. Inclusion of inulin or combined supplementation of inulin and cellulose to sow diets improved both maternal and offspring health [10, 16], which led us to hypothesize whether the beneficial effects of dietary fiber on reproductive performance of sows could be related to its microbial and immune modulation actions. In the current study, gestation diets of sows were supplemented with purified dietary fiber guar gum and cellulose, to investigate the effects of dietary fiber levels on reproductive performance and the associated changes in microbial compositions and immune function.

## Materials and methods

### Animals and experimental design

This study was conducted in Guangxi Guilin Group Swine Breeding CO. LTD., where approximately 6000 sows were maintained. A total of 210 Landrace × Yorkshire sow (parities 3–6) with similar farrowing time were allocated to six dietary treatment groups (*n* = 35) with each sow as a replicate in a completely randomized design. Sows were artificially inseminated twice with pooled semen from three Duroc boars. To minimize the variations of insemination operations, all insemination was conducted by only one experienced technician. Control (CON) diet was corn-soybean meal-based without wheat bran to contain 12.5% dietary fiber (Table 1). Wheat bran was added to the CON diet to increase concentration of dietary fiber (DF1, 17.4% dietary fiber), and a FIBER MIX (Guangxi Shangda Tech Co. LTD, Nanning, China), which contains guar gum and cellulose at the ratio of 1:4, was included at the levels of 1% (DF2), 2% (DF3) 3% (DF4) and 4% (DF5) to partially replace the wheat bran in the DF1 diet (Table 1). All diets were formulated to meet or exceed the nutrient requirements of gestating sows with an anticipated total litter size of 12.5 and a gestation weight gain of 50 kg as recommended by NRC (2012) [17]. In this study, the definition of DF is different from those of crude fiber (CF) or neutral detergent fiber (NDF). DF, the sum of soluble fiber (SF) and insoluble fiber (IDF), was measured by enzymatic-gravimetric method with minor modification [18] . In brief, 1 g feed sample was stirred with a 40-mL MES-TRIS buffer solution (Sigma-Aldrich, Saint Louis, USA) on a magnetic stirrer. 50 μL heat-stable α-amylase solution (A3306, Sigma-Aldrich) was added to the mixture and was incubated in a 95–100 °C water bath for 15 min with continuous agitation, followed by protease solution treatment (P3910, Sigma-Aldrich) for 30 min at 60 °C. After adjusting pH to 4.0–4.7, 300 μL amyloglucosidase solution (A9913, Sigma-Aldrich) was added to the solution which was incubated for 30 min at 60 °C with constant agitation. After hydrolysis, the IDF residues were obtained by filtration on a crucible with acid-washed wet and redistribute Celite (C8656, Sigma-Aldrich), and the filtrate was collected by adding 95% ethanol prewarmed at 60 °C to form the SDF precipitate. Guar gum is water-soluble and easy to be fermented in the gut by the microbiota. Cellulose is insoluble to water and is not easy to be fermented by the gut microbiota.

**Table 1:** Ingredients and nutrient compositions of gestation diets (as-fed basis)

	CON^c^	DF1	DF2	DF3	DF4	DF5
Ingredient, %						
Corn	79	64	64	64	64	64
Soybean meal	17	14	14	14	14	14
Wheat bran	–	18	16.4	14.8	13.2	11.6
Fish meal	–	–	0.3	0.6	0.9	1.2
Soybean oil	–	–	0.3	0.6	0.9	1.2
FIBER MIX^a^	–	–	1	2	3	4
Premix^b^	4	4	4	4	4	4
Total	100	100	100	100	100	100
Calculated nutrient composition						
DE, Mcal/kg	3.25	3.05	3.05	3.05	3.05	3.05
Crude protein, %	13.5	13.9	13.8	13.8	13.7	13.7
Total lysine, %	0.64	0.64	0.64	0.65	0.65	0.65
Total threonine, %	0.51	0.51	0.51	0.51	0.51	0.51
Total methionine, %	0.23	0.23	0.23	0.23	0.23	0.23
Total tryptophan, %	0.15	0.16	0.16	0.16	0.16	0.16
SID lysine, %	0.55	0.53	0.54	0.54	0.55	0.56
SID methionine, %	0.21	0.21	0.21	0.21	0.21	0.21
SID threonine, %	0.43	0.41	0.42	0.42	0.42	0.42
SID tryptophan, %	0.13	0.14	0.13	0.13	0.13	0.13
Calcium, %	0.78	0.78	0.79	0.8	0.81	0.82
Phosphorus, %	0.52	0.63	0.62	0.61	0.61	0.56
DF, %	12.5	17.4	17.7	18.1	18.4	18.8
CF, %	2.9	3.9	4.6	5.2	5.9	6.6
NDF, %	11.0	15.4	15.7	15.9	16.1	16.3
SF, %	1.57	2.02	2.16	2.29	2.42	2.56
ISF, %	10.91	15.33	15.56	15.79	16.01	16.24

^a^ FIBER MIX consisted of guar gum and cellulose at the ratio of 1:4, obtained from Guangxi Shangda Tech Co. LTD, Nanning, China

^b^Provided per kg of diet: Fe, 100 mg as ferrous sulfate; Cu, 6.6 mg as copper sulfate; Mn, 30 mg as manganese sulfate; Se, 0.15 mg as sodium selenite; Zn, 100 mg as zinc sulfate; I, 0.6 mg as KI potassium iodide; 6608 IU of vitamin A, 1652 IU of vitamin D_3_, 27.5 IU of vitamin E, 4.4 mg of vitamin K, 1.66 mg of thiamine, 6.6 mg of riboflavin, 40 mg of niacin, 25 mg of pantothenic acid, 33 μg of vitamin B_12_, 0.8 mg of pyridoxine, 1.5 mg of folic acid, 0.22 mg of biotin and 583 mg of choline. SF and ISF were analyzed value according to AOAC 991.43 with minor modification. DF = SF + ISF

^c^CON, basal corn-soybean diet; DF1, wheat bran-rich diet; DF2, DF3, DF4 and DF5 were wheat bran diet supplemented with 1%, 2%, 3% and 4% of FIBER MIX at the expense of wheat bran

Sows in DF1, DF2, DF3, DF4, and DF5 groups were fed 2.0 kg/d diets from gestation day 1 to day 7, 2.4 kg/d diet from day 8 to day 89 and 3.2 kg/d diet from day 90 to parturition. The CON sows were fed 2.0 kg/d diets from gestation day 1 to day 7, 2.2 kg/d diets from day 8 to day 89 and 3.1 kg/d diets from day 90 to parturition. Finally, all sows consumed similar DE, amino acids and other nutrients per day except for different levels of dietary fiber. Sows were housed in individual gestation stalls (2.20 m × 0.65 m) from day 1 to day 106 of gestation. On day 107 of gestation, sows were moved to individual farrowing pen and continued to consume their gestation diets. Sows were fed twice daily at 08:00 h and 16:00 h and had free access to water throughout the experiment. The average ambient temperature in the gestation house was at 22–26 °C.

During lactation, all sows were fed a common diet providing 3380 kcal DE/kg and 18% crude protein (Table 2). Sows were fed 0.5 kg of lactation diet on the day of parturition and 2 kg on day 2 of lactation and then the ration was increased by 0.5 kg/d from day 3 to day 5 of lactation. Then all sows had free access to feed throughout lactation. Newborn piglets were cross-fostered within each dietary group 24 h after farrowing. Routine piglet management processing procedures of the farm including intramuscular injection of 1 mL iron dextran shots, tail docking, cutting and disinfection of navel cords were performed within 72 h after birth. Piglets were not allowed to receive creep feed but had access to the dam’s feed during lactation. Each farrowing unit was equipped with a 250-W heat lamp to maintain a constant temperature for each litter after birth for 48 h, and thereafter the 150-W heat lamps were used to provide heat until weaning. The farrowing room was maintained at 24 ± 2 °C during lactation. Piglets were weaned at day 18 (± 1 d) of lactation. During lactation, feed refusals were weighed daily, and feed intake of each sow was calculated. The residual feed contaminated by water was dried before weighing.

**Table 2:** Ingredients and nutrient compositions of lactation diets (as-fed basis)

Ingredients	%
Corn	66.36
Soybean meal	25.00
Soybean oil	2.00
Fish meal	3.00
Limestone	0.40
CaHPO_4_	1.30
Salt	0.40
*L*-lysine (78%)	0.11
*L*-threonine (98.5%)	0.05
Choline chloride (50%)	0.15
Premix^a^	1.00
Calculated nutrient content	
DE, Mcal/kg	3.38
Crude protein, %	18.0
Calcium, %	0.80
Phosphorus, %	0.65
Total lysine, %	1.05
Total methionine, %	0.30
Total threonine, %	0.75

^a^Provided per kg of diet: Fe, 120 mg as ferrous sulfate; Cu, 20 mg as copper sulfate; Mn, 60 mg as manganese sulfate; Se, 0.3 mg as sodium selenite; Zn, 60 mg as zinc sulfate; I, 1 mg as KI potassium iodide; 7000 IU of vitamin A, 2000 IU of vitamin D_3_, 200 IU of vitamin E, 5 mg of vitamin K, 4 mg of thiamine, 10 mg of riboflavin, 5 mg of pyridoxine, 30 μg of vitamin B_12_, 50 mg of niacin, 30 mg pantothenic acid, 5 mg of folic acid and 0.22 mg of biotin

### Measurements of reproductive performance

Backfat thickness was measured at 65 mm to the left side of the dorsal mid-line at the level of the last rib (P2) at days 0, 30, 60 and 90 of gestation, farrowing and weaning, using a Digital Backfat Indicator (Renco Lean-Meater; Renco Corporation, Minneapolis, MN, USA). Feces of sows were scored by judging the appearance of feces 1 h after the morning meal on days 1, 10 20, 30 and 40 of gestation using the following scale: 1 = dry and hard, 2 = firm, 3 = normal stool with no evidence of compaction, 4 = loose stool, 5 = watery as described by Darroch et al. [19]. Farrowing was attended and farrowing duration, the total number of pigs born, born alive, stillborn or mummified were recorded. The birth weight of piglets was weighed 1 h after the last born of piglets. Besides, the uniformity of newborns was determined using the intra-litter coefficient of variation (CV). BW of each piglet and litter weaning weight were recorded at the end of lactation. Estrus detection with a fence-line boar exposure was conducted by only one experienced stockperson the day after their weaning, and the appearance of standing heat under applied back pressure was used as an important criterion to establish the onset of post-weaning estrus, and weaning-to-estrous interval (WEI) of the post-weaning sow was recorded.

### Collection of blood and fecal samples

Ten mL blood samples from ear vein were collected from 6 sows per group at 12:00 h on days 30, 110 of gestation and at farrowing for analyzing cytokine concentrations. On the day of birth and weaning, 5 mL blood samples were obtained from the jugular vein of piglets from six sows per treatment group with each litter providing one piglet, whose body weight was close to the within-litter average weight. Blood samples were centrifuged at 3,000×*g* for 30 min at 4 °C. The serum was transferred to 200 μL centrifuge tubes and stored at − 20 °C until analysis. Fresh feces of six sows per treatment which were subjected to blood sampling, were collected and kept on ice until transferring them to a freezer at − 80 °C within 10 min in the morning immediately after defecation at days 30, 60, 90 and 110 of gestation, respectively.

### Colostrum and milk sampling

Colostrum was collected by hand-milking 1 h after the birth of the last piglet without oxytocin administration. Milk was collected at day 7 ± 1 of lactation. Colostrum and milk samples were collected from anterior, middle and posterior mammary glands and pooled together, and were immediately filtered through gauze and stored at − 20 °C.

### Fecal SCFAs and microbial analyses

The SCFAs concentrations were measured with a previously described method with minor modifications [20]. Briefly, 1 g of fecal sample was suspended in 1.5 mL of distilled water and placed at 4 °C in a refrigerator for 30 min. Afterward, the sample was centrifuged (15,000×*g*) at 4 °C for 15 min. The 1 mL supernatant was transferred and mixed with a 0.2-mL 25% (w/v) metaphosphoric acid and 23.3 μL crotonic acid (210 mmol/L, internal standard). After 30 min at 4 °C, the sample was centrifuged (15,000×*g*) again at 4 °C for 10 min. The supernatant (0.3 mL) was transferred and mixed with 0.9 mL methanol before centrifuge. Afterward, the sample was centrifuged (10,000×*g*) at 4 °C for 10 min before filtered through 0.22 μm filter membrane. An aliquot of the filtrate (1 μL) was analyzed using gas chromatography (Varian CP-3800 GC, USA).

The microbial diversity was determined as previously described [20]. Briefly, microbial DNA was extracted from 0.25 g of thawed stool samples using a QIAamp DNA Stool Mini Kit (Qiagen, Hilden, Germany) according to the manufacturer’s protocol. The integrity of the extracted genomic DNA was determined by electrophoresis on a 1% (w/v) agarose gel. The quality and quantity of DNA were measured using a NanoPhotometer® spectrophotometer (IMPLEN, CA, USA). An absorption ratio (260/280 nm) within 1.8–2.0 was deemed to be of sufficient purity to be used for subsequent analyses. DNA samples were sent to a commercial service provider (Novogene, Beijing, China) for amplicon pyrosequencing on an Illumina MiSeq platform according to the manufacturer’s instruction. A total amount of 1 μg DNA per sample was used in the preparation of amplicons for the high-throughput sequencing of microbial 16S rRNA. The V4 hypervariable region of the 16S rRNA gene was amplified using universal primers 515F and 806R (5′-GTGCCAGCMGCCGCGGTAA-3′and 5′-GGACTACHVGGGTWTCTAAT-3′).

### Cytokines analysis

Serum concentrations from sows and piglets were detected for interleukin-6 (IL-6), interleukin-10 (IL-10), tumor necrosis factor-α (TNF-α) and interferon-γ (INF-γ) using the enzyme-linked immunosorbent assay (ELISA) Kit (Jiancheng Institute of Biological Technology, Nanjing, China). All of the cytokine analysis was referred to the manufacturer’s instructions, and each sample was analyzed in duplicates.

### Colostrum and milk composition

Frozen colostrum and milk samples were thawed at 4 °C, and 10 mL of each sample was used to analyze the milk compositions as previously described [21]. Lipids, protein, lactose and total solids of colostrum and milk were measured by Milk Composition Analyzer (Milk-Yway-CP2, Beijing, China).

### Statistical analysis

The sow was the experimental unit for all the parameters analyzed. The normal distribution of data was verified by a Kolmogorov–Smirnov test before analysis. Differences in the data including sow performance, litter performance, milk compositions and serum cytokine concentrations of piglets were applied to the following model using the GLM procedure of SAS 9.4 (SAS Institute, Inc., Cary, NC, USA):

$$ {\mathrm{Y}}_{\mathrm{i}}=\upmu +{\upalpha}_{\mathrm{i}}+{\upvarepsilon}_{\mathrm{i}}, $$

where Y_i_ is the response variable, μ is the overall mean, α_i_ is the fixed effect of dietary fiber level and ε_i_ is the residual error. Data of reproductive performance (total born, mean BW of piglet at birth, mean litter weight at birth, stillborn rate, intralitter CV, duration of farrowing and ADFI during lactation) were analyzed using parity as a covariate, and born alive were analyzed using both parity and total born as covariates following the model above. Studies of the relationship between dietary fiber levels (CF, NDF, DF, SF, and ISF) and reproductive performance were analyzed by Pearson correlation, and Orthogonal Linear contrasts (OPC) analysis was used to test the linear and quadratic effects of dietary fiber levels on parameters of sows and piglets. Data of relative abundance at phylum and genus level were log-transformed before statistical analysis. Concentrations of serum cytokine concentrations of sows, SCFAs in feces, scores of feces, alpha diversity index and log-transformed relative abundances at different taxonomic levels were applied to the following model using MIXED procedure of SAS:

$$ {\mathrm{Y}}_{\mathrm{i}\mathrm{j}\mathrm{k}}=\upmu +{\upalpha}_{\mathrm{i}}+{\upbeta}_{\mathrm{j}}+{\left(\upalpha \upbeta \right)}_{\mathrm{i}\mathrm{j}}+{\mathrm{t}}_{\mathrm{k}}+{\upvarepsilon}_{\mathrm{i}\mathrm{j}\mathrm{k},} $$

where Y_ijk_ is the response variable, μ is the overall mean, α_i_, and β_j_ are the fixed effects of dietary fiber level and gestation stage, respectively. (αβγ)_ij_ is the interaction among fixed effects, and it is the random effect of the sow to account for repeated measurements within sow and ε_ijk_ is the residual error. When the main effects were significant, the means were compared using Turkey’s test. Pearson’s correlation was used to study the relationship between gestation stage, SCFAs, serum parameters and dietary fiber levels and the relative abundance of fecal microbial phylum and genus. Meanwhile, Pearson correlations between the gut microbiota and reproductive performance and serum cytokines of sows were performed. The results were expressed as mean ± largest SEM in tables and as means ± SEM in figures. A statistical difference was declared at *P <* 0.05, whereas *P <* 0.10 was considered a tendency.

## Results

### Sow and litter performance

A total of 210 sows (*n* = 35) were selected at the start of the experiment, but 20 sows were excluded from data analysis due to conception failure (9 sows), illness (5 sows), lameness (3 sows) and other reasons (3 sows). A final number of 30, 33, 32, 32, 31 and 32 sows in CON, DF1, DF2, DF3, DF4 and DF5 groups, respectively, was used for statistical analysis of reproductive parameters. The backfat thickness of sows at days 30, 60, 90 of gestation and at farrowing was not affected by dietary fiber levels (*P* > 0.05, Table 3). The number of total born piglets tended to be affected by dietary treatment (*P* = 0.071, Table 3), and was linearly increased by dietary fiber levels (*P* <  0.01, Table 3). The mean BW of the piglet at birth, mean litter weight at birth, stillborn rate, and intralitter CV were not affected by dietary treatment (*P* > 0.01, Table 3). The duration of the farrowing was not affected by dietary fiber levels (*P* > 0.05, Table 3). Pearson’s correlation analysis showed that the number of total born piglets and piglets born alive, but not mean BW of piglet at birth, mean litter weight at birth, stillborn rate or intralitter CV, were positively correlated with CF, NDF, TDF, SF or ISF levels (*P* < 0.05 or *P* < 0.01, Table 4).

**Table 3:** Effects of dietary fiber levels during gestation on sow performance

Item	CON	DF1	DF2	DF3	DF4	DF5	SEM	*P-*value		
								Diets	Linear	Quadratic
Number of sow	30	33	32	32	31	32				
Sow parity	3.1	3.4	3.5	3.5	3.7	3.8	0.12	0.42	0.094	0.93
Sow BF thickness, mm										
At breeding	14.3	15.0	13.8	13.7	14.7	14.9	0.24	0.48	0.40	0.79
Day 30	15.1	15.7	14.1	14.3	15.6	15.1	0.22	0.19	0.72	0.98
Day 60	16.1	16.5	15.4	15.7	16.6	16.6	0.22	0.48	0.19	0.82
Day 90	16.6	16.8	16.3	17.2	17.6	17.2	0.22	0.52	0.25	0.12
Farrowing	17.5	17.2	16.8	17.4	17.8	17.6	0.23	0.86	0.081	0.15
BF gain during gestation, mm	3.3	2.2	3.0	3.7	3.2	2.7	0.13	0.12	0.19	< 0.01
Number of total born	12.9	13.7	13.8	14.1	13.9	14.8	0.17	0.071	< 0.01	0.20
Number of born alive	12.1	12.4	12.9	12.8	13.2	13.9	0.16	0.19	< 0.01	0.12
Mean BW at birth, kg	1.44	1.41	1.42	1.45	1.44	1.39	0.013	0.78	0.29	0.54
Litter weight at birth, kg	17.4	17.5	18.3	18.6	19.1	19.3	0.27	0.23	0.95	0.64
Stillborn rate, %	5.80	7.99	6.54	7.76	4.82	6.03	2.38	0.10	0.76	0.49
Intralitter CV, %	20.6	18.5	18.5	19.7	18.2	18.71	0.42	0.56	0.44	0.81
Duration of farrowing, min	234.1	285.5	290.8	296.8	293.0	289.9	11.6	0.65	0.10	0.17

Data are expressed as mean ± largest SEM. CON, basal corn-soybean diet; DF1, wheat bran-rich diet; DF2, DF3, DF4 and DF5 were wheat bran diet supplemented with 1%, 2%, 3% and 4% of FIBER MIX at the expense of wheat bran; *BW* body weight, *BF* backfat thickness

**Table 4:** Sow performance correlated by Pearson’s correlation to different fiber components in gestation diets

	CF	NDF	TDF	SF	ISF
Number of total born	0.213**	0.195**	0.201**	0.217**	0.177*
Number of born alive	0.236**	0.175*	0.184*	0.225**	0.204**
Mean BW of piglet at birth, kg	−0.042	−0.041	−0.043	−0.043	− 0.006
Mean litter weight at birth, kg	−0.014	− 0.023	−0.023	− 0.018	−0.023
Stillborn rate, %	0.046	0.045	0.046	0.049	0.044
Intralitter CV, %	−0.033	−0.029	− 0.031	−0.032	− 0.030

* denotes *P* < 0.05 and ** denotes *P* < 0.01

After delivery, 3, 3, 2, 2, 1 and 2 sows in CON, DF1, DF2, DF3, DF4 and DF5, respectively, were removed due to puerperal fever (3 sows), udder problems (4 sows), lameness (3 sows) and other reasons (3 sows). The ADFI of sows during lactation were linearly increased by the dietary fiber levels during gestation (*P* < 0.05, Table 5), and sows in the DF2, DF3 and DF5 treatments had a greater ADFI during lactation than the sows in the CON group (*P* < 0.05, Table 5). The average weaning body weight of piglets, backfat thickness at weaning and backfat loss during lactation were not affected by diets (*P* > 0.05, Table 5). The weaning-to-estrus intervals of sows were not affected by diets (*P* > 0.05, Table 5).

**Table 5:** Effects of dietary fiber levels during gestation on growth performance of suckling piglets

Items	CON	DF1	DF2	DF3	DF4	DF5	SEM	*P-* value		
								Diets	Linear	Quadratic
Number of sows	27	30	30	30	30	30		–	–	–
Litter size										
After cross-foster	12.2	12.7	12.5	11.9	12.0	11.9	0.10	0.31	0.37	0.19
At weaning	11.2	11.4	11.6	11.0	11.0	11.0	0.11	0.52	0.36	0.07
Mean BW of piglet, kg										
After cross-foster	1.72	1.64	1.69	1.71	1.70	1.76	0.08	0.61	0.62	0.11
At weaning	6.09	5.98	6.20	6.31	6.31	6.12	0.04	0.20	0.38	0.13
Litter weight, kg										
After cross-foster	20.8	20.8	21.1	20.2	20.3	20.6	0.24	0.91	0.34	0.51
At weaning	67.8	68.0	71.5	70.0	69.0	66.9	0.55	0.26	0.11	0.35
Daily BW gain of piglets, g/d	233.5	228.4	237.1	242.3	242.6	229.9	2.35	0.31	0.071	0.33
Mean litter weight gain, kg	49.97	49.21	51.68	50.54	50.54	48.45	0.26	0.28	0.02	0.62
BF thickness at weaning, mm	14.8	14.5	14.1	14.6	14.8	15.3	0.77	0.22	0.032	0.96
BF loss during lactation, mm	2.8	2.7	2.8	2.6	2.9	2.4	0.14	0.94	0.45	0.048
ADFI, kg	4.74^b^	5.07^ab^	5.24^a^	5.33^a^	5.00^ab^	5.20^a^	0.06	0.042	0.011	0.48
WEI, d	4.8	4.5	4.1	4.2	4.2	4.2	0.13	0.53	0.49	0.65

Data are expressed as mean ± largest SEM. ^a, b^ denote *P <* 0.05. CON, basal corn-soybean diet; DF1, wheat bran-rich diet; DF2, DF3, DF4 and DF5 were wheat bran diet supplemented with 1%, 2%, 3% and 4% of FIBER MIX at the expense of wheat bran. *ADFI* average daily feed intake, *WEI* weaning to estrus interval

### Colostrum and milk compositions

The compositions of colostrum and milk were shown in Table 6. The lipid contents of colostrum were affected linearly by dietary fiber levels (*P* < 0.05, Table 6), and sows fed the DF2 diet had higher lipid concentrations in colostrum than those fed the CON and DF5 diets (*P* < 0.05, Table 6). The concentrations of protein, lactose and total solids in colostrum were not affected by diets (*P* > 0.05, Table 6). No differences of lipids, protein, lactose and total solids concentrations were found in milk by diets (*P* > 0.05, Table 6).

**Table 6:** Effects of dietary fiber levels during gestation on colostrum and milk compositions of sows

Item	CON	DF1	DF2	DF3	DF4	DF5	SEM	*P*-value		
								Diets	Linear	Quadratic
Colostrum, %										
Lipids	4.1^b^	6.0^ab^	6.8^a^	5.7^ab^	5.1^ab^	3.9^b^	0.80	0.022	0.043	0.10
Protein	7.3	7.1	8.2	7.1	7.5	7.0	0.48	0.18	0.31	0.56
Lactose	11.3	10.6	12.8	10.3	11.3	10.6	0.96	0.23	0.38	0.96
Total solids	19.8	19.1	22.0	19.2	20.3	18.9	1.26	0.18	0.31	0.57
Milk, %										
Lipids	7.4	7.1	7.0	7.2	6.6	8.6	1.26	0.62	0.14	0.34
Protein	3.3	3.5	3.7	3.8	3.9	3.7	0.40	0.54	0.65	0.06
Lactose	4.7	5.0	5.5	5.4	6.3	5.2	0.59	0.18	0.85	0.02
Total solids	8.9	9.4	10.0	10.1	10.6	9.8	1.10	0.56	0.70	0.07

Data are expressed as mean ± largest SEM. Sows were regarded as the experimental units, *n* = 6 for each treatment. ^a, b^ denote *P <* 0.05. CON, basal corn-soybean diet; DF1, wheat bran-rich diet; DF2, DF3, DF4 and DF5 were wheat bran diet supplemented with 1%, 2%, 3% and 4% of FIBER MIX at the expense of wheat bran

### Serum concentrations of cytokines

Serum concentrations of cytokines of sows were presented in Table 7. The serum concentrations of IL-10, TNF-α and IFN-γ of sows were significantly affected by the stage of gestation (*P <* 0.01 or *P <* 0.05). The serum concentrations of IFN-γ of sows tended to be affected by the dietary fiber levels (*P =* 0.070). The concentrations of IL-10 and TNF-α on day 30 of gestation were affected linearly by dietary fiber levels (*P* < 0.01). Serum concentrations of cytokines of piglets were presented in Table 7. Serum concentrations of IL-6, IL-10, TNF-α and IFN-γ of piglets at birth and at weaning were not affected by dietary fiber levels, except for both linear and quadratic effects of dietary fiber levels on serum TNF-α concentrations of piglets at birth (*P <* 0.05), and newborn piglets of sows receiving the DF5 diets had lower serum TNF-α concentrations (− 12%; *P* < 0.05) compared with piglets of sows receiving the CON diet.

**Table 7:** Effects of dietary fiber levels during gestation on serum concentrations of cytokines in sows

Item	CON	DF1	DF2	DF3	DF4	DF5	SEM	*P-*value					
								Stage	Diets	S × D	Diets	Linear	Quadratic
Sows													
IL-6, ng/L								0.19	0.33	0.99	–	–	–
D 30 of gestation	125.9	105.7	119.4	116.8	105.7	111.3	7.3	–	–	–	0.95	0.81	0.61
D 110 of gestation	136.8	102.4	145.8	124.1	120.5	114.8	10.4	–	–	–	0.88	0.79	0.88
At farrowing	117.6	73.1	107.4	89.7	107.1	92.7	9.3	–	–	–	0.78	0.77	0.88
IL-10, ng/L								0.031	0.83	0.93	–	–	–
D 30 of gestation	152.7^b^	171.7^ab^	197.7^ab^	197.6^ab^	209.8^ab^	248.1^a^	8.3	–	–	–	0.041	< 0.01	0.13
D 110 of gestation	230.2	228.6	219.9	247.4	254.3	223.1	14.9	–	–	–	0.98	0.85	0.54
At farrowing	173.6	166.9	192.4	160.9	192.1	172.3	21.8	–	–	–	0.99	0.96	0.87
TNF-α, ng/L								< 0.01	0.72	0.65	–	–	–
D 30 of gestation	28.8^a^	28.4^a^	26.8^ab^	23.9^ab^	22.5^ab^	20.4^b^	0.8	–	–	–	0.042	< 0.01	0.064
D 110 of gestation	22.1	20.0	19.3	21.6	23.3	18.9	1.2	–	–	–	0.87	0.51	0.61
At farrowing	20.5	21.3	20.6	19.9	19.4	20.7	1.2	–	–	–	0.99	0.96	0.72
IFN-γ, ng/L								< 0.01	0.070	0.99	–	–	–
D 30 of gestation	147.0	119.4	137.3	111.5	120.2	107.3	8.4	–	–	–	0.73	0.37	0.41
D 110 of gestation	133.6	108.4	147.0	101.3	123.8	120.5	9.8	–	–	–	0.84	0.85	0.68
At farrowing	103.7	66.1	84.8	64.9	74.8	67.4	4.8	–	–	–	0.22	0.045	0.017
Piglets													
At birth													
IL-6, ng/L	165.2	126.1	151.2	130.6	149.6	156.4	4.5	–	–	–	0.20	0.34	0.13
IL-10, ng/L	52.6	53.9	52.7	54.4	53.5	55.6	0.9	–	–	–	0.95	0.79	0.48
TNF-α, ng/L	38.3^a^	36.6^ab^	37.5^bc^	35.1^ab^	34.1^ab^	33.9^b^	0.4	–	–	–	0.033	< 0.01	0.023
IFN-γ, ng/L	106.9	95.5	94.8	100.6	103.5	96.9	1.9	–	–	–	0.41	0.42	0.84
At weaning													
IL-6, ng/L	171.4	167.7	152.2	161.6	179.8	154.8	10.3	–	–	–	0.97	0.66	0.81
IL-10, ng/L	54.3	93.5	72.6	102.5	91.3	80.0	8.6	–	–	–	0.70	0.88	0.23
TNF-α, ng/L	49.4	58.7	55.9	49.3	58.4	51.1	2.9	–	–	–	0.87	0.71	0.58
IFN-γ, ng/L	120.5	126.3	127.8	131.2	148.2	110.9	5.8	–	–	–	0.65	0.35	0.12

Data are expressed as mean ± largest SEM. Sows were regarded as the experimental units, *n* = 6 for each treatment. ^a, b, c^ denote *P <* 0.05. CON, basal corn-soybean diet; DF1, wheat bran-rich diet; DF2, DF3, DF4 and DF5 were wheat bran diet supplemented with 1%, 2%, 3% and 4% of FIBER MIX at the expense of wheat bran

### Changes of fecal SCFAs and scores

SCFAs concentrations in the feces of gestating sows fed different dietary fiber levels were shown in Table 8. Gestation stage had noteworthy effects on concentrations of acetate, propionate, butyrate and total SCFAs in feces (*P <* 0.01). The concentrations of acetate, propionate, butyrate and total SCFAs in feces of sows were greater at the days 90 and 110 of gestation than those at days 30 and 60 of gestation (*P <* 0.01). Concentrations of acetate and butyrate in feces of sows were linearly affected by dietary fiber levels (*P <* 0.01). Concentrations of acetate, propionate and total SCFAs in feces were altered by the interaction between diets and stage (*P <* 0.05).

**Table 8:** Effect of dietary fiber levels on fecal short-chain fatty acids concentrations of gestating sows

Item	Gestation stage				Diets						SEM	*P*-value				
	Day 30	Day 60	Day 90	Day 110	CON	DF1	DF2	DF3	DF4	DF5		Stage	Diets	S × D	Linear	Quadratic
Acetate, μmol/g	59.4^b^	57.9^b^	74.9^a^	67.7^ab^	65.5	65.9	56.0	64.6	63.5	74.0	5.1	< 0.01	0.15	0.041	0.032	0.35
Propionate, μmol/g	20.5^b^	20.6^b^	27.9^a^	26.4^a^	24.8	27.1	21.1	22.7	24.2	22.9	2.4	< 0.01	0.16	0.022	0.45	0.36
Butyrate, μmol/g	10.3^b^	9.8^b^	13.9^a^	13.3^a^	10.9^b^	12.3^ab^	9.2^b^	10.7^b^	12.2^ab^	15.3^a^	1.1	< 0.01	< 0.01	0.13	< 0.01	0.52
Total SCFAs, μmol/g	90.2^bc^	88.3^c^	116.7^a^	107.4^ab^	101.2	105.3	86.3	98.0	99.9	112.3	8.3	< 0.01	0.15	0.042	0.071	0.36

Data are expressed as mean ± largest SEM. Sows were regarded as the experimental units, *n* = 6 for each treatment. ^a, b, c^ denote *P <* 0.05. CON, basal corn-soybean diet; DF1, wheat bran-rich diet; DF2, DF3, DF4 and DF5 were wheat bran diet supplemented with 1%, 2%, 3% and 4% of FIBER MIX at the expense of wheat bran

Scores of the feces of gestating sows fed different dietary fiber levels were shown in Table 9. The fecal scores of gestations were significantly affected by the gestation stage (*P <* 0.01), and the scores of feces on sows at day 1 of gestation were greater than those on days 10, 20, 30 and 40 of gestation. The fecal scores of gestations were significantly affected by dietary treatment (*P <* 0.01), which were affected linearly and quadratically by dietary fiber levels.

**Table 9:** Effect of dietary fiber levels on fecal scores of gestating sows

Item	Gestation stage					Diets						SEM	*P*-value				
	Day 1	Day 10	Day 20	Day 30	Day 40	CON	DF1	DF2	DF3	DF4	DF5		Stage	Diets	S × D	Linear	Quadratic
Scores	2.93^a^	2.68^b^	2.69^b^	2.57^b^	2.71^b^	2.42^b^	2.70^ab^	2.74^a^	2.73^a^	2.83^a^	2.86^a^	0.07	< 0.01	< 0.01	0.84	< 0.01	< 0.01

Data are expressed as mean ± largest SEM. Sows were regarded as the experimental units, *n* = 30, 33, 32, 32, 31 and 32 for CON, DF1, DF2, DF3, DF4 and DF5 at each stage of gestation, respectively. ^a, b^ denote *P <* 0.05. CON, basal corn-soybean diet; DF1, wheat bran-rich diet; DF2, DF3, DF4 and DF5 were wheat bran diet supplemented with 1%, 2%, 3% and 4% of FIBER MIX at the expense of wheat bran

### Changes of fecal microbiota

At the phylum level, Spirochaetes and Cyanobacteria were negatively correlated with DF levels, whereas Verrucomicrobia were positively correlated with DF levels (Supplementary Table 1). At the genus level, six genera were affected by dietary treatment (Supplementary Table 2). *Streptococcus* in Firmicutes, *Prevotellaceae_NK3B31_group, Prevotella_1, Prevotella_9* in Bacteroidetes and *Succinivibrio* in Proteobacteria were negatively correlated with dietary DF levels, whereas *Prevotellaceae_UCG-001* (p: Bacteroidetes) was positively correlated with dietary DF levels (*P <* 0.01, Supplementary Table 2).

**Fig. 1:**
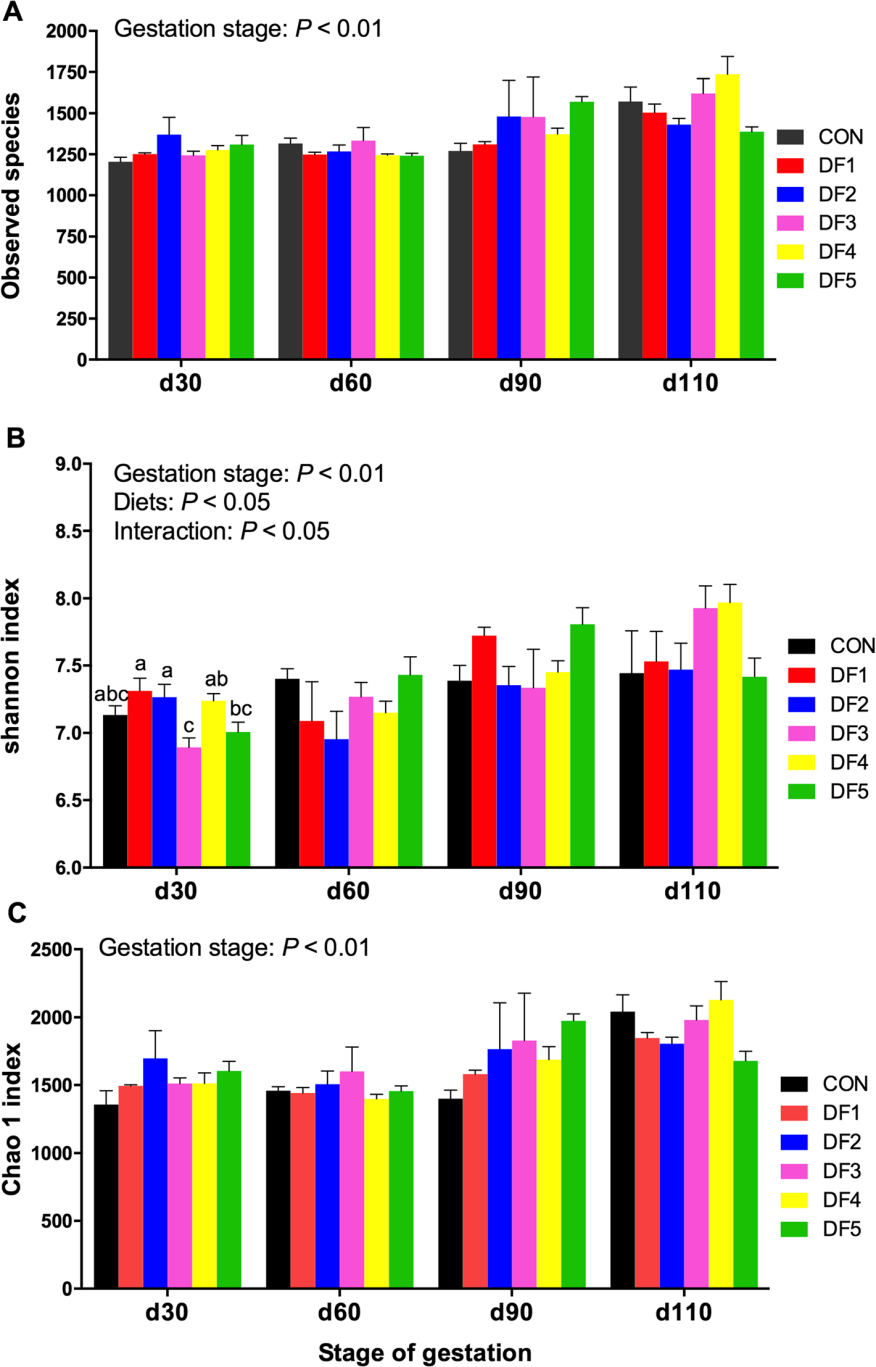
Microbiota alpha-diversity in feces of sows fed different dietary fiber levels at different gestation stages. Sow was regarded as the experimental units (*n* = 6). **a** Observed species; **b** Shannon index and **c** Chao 1 index. CON, basal corn-soybean diet; DF1, wheat bran-rich diet; DF2, DF3, DF4 and DF5 were wheat bran diet supplemented with 1%, 2%, 3% and 4% of FIBER MIX at the expense of wheat bran. Columns with different letters ^a,b,c^ denote *P* < 0.05

The average raw read and effective tags of all the samples were 89,229 and 84,857, respectively. To assess fecal microbial alpha diversity, the observed species, Shannon and Chao 1 index were calculated and presented in Fig. 1. Observed species, Shannon and Chao 1 index were significantly affected by the gestation stage (*P <* 0.01, Fig. 1a-c), which were increased with the progression of gestation and reached the peak on gestation day 110. There was an interactive effect between diet and gestation stage on the Shannon index (*P <* 0.05).

The relative abundances at the phylum level in feces of sows during gestation were presented in Table 10. Firmicutes and Bacteroidetes were the two dominant phyla consisting about 90% of the fecal microbiota. Microbiota of Firmicutes, Bacteroidetes, Euryarchaeota, Proteobacteria, Spirochaetes, Tenericutes, Cyanobacteria, Actinobacteria, Acidobacteria, Planctomycetes, Verrucomicrobia, Fibrobacteres, and the ratios of Firmicutes/Bacteroidetes were significantly affected by gestation stage (*P* < 0.05 or *P* < 0.01). The Acidobacteria abundance was linearly affected by dietary fiber levels (*P <* 0.05), and the abundances of Cyanobacteria and Verrucomicrobia were quadratically affected by dietary fiber levels (*P <* 0.05). Bacteroidetes, Proteobacteria, Spirochaetes, Acidobacteria, Planctomycetes and Fibrobacteres abundances and ratios of Firmicutes/Bacteroidetes were affected by the interaction between gestation stage and diets (*P <* 0.01, Table 10).

**Table 10:** The relative abundances of microbiota at phyla level and Firmicutes/Bacteroidetes ratio in feces of sows during gestation (%)

Item	Gestation Stage				Diets						SEM	*P*-value				
	Day 30	Day 60	Day 90	Day 110	CON	DF1	DF2	DF3	DF4	DF5		Stage	Diets	S × D	Linear	Quadratic
Firmicutes	80.15^a^	73.98^b^	67.09^a^	64.57^a^	71.15	74.71	71.77	70.96	69.26	70.82	3.6	< 0.01	0.29	0.031	0.66	0.47
Bacteroidetes	10.67^a^	15.76^b^	21.09^a^	20.63^a^	16.66	15.64	18.00	18.00	17.56	16.35	1.9	< 0.01	0.41	< 0.01	0.80	0.43
Euryarchaeota	1.06^b^	2.02^a^	2.09^a^	1.86^ab^	2.47	0.70	1.72	2.00	1.61	2.06	0.7	0.02	0.33	< 0.01	0.56	0.57
Proteobacteria	1.21^b^	1.25^b^	2.13^b^	5.01^a^	1.73	1.65	1.56	2.31	3.81	3.34	1.5	< 0.01	0.17	0.043	0.12	0.17
Spirochaetes	2.06^b^	2.34^ab^	3.24^a^	2.66^ab^	3.39	2.33	2.57	2.38	2.3	2.47	0.5	0.02	0.08	0.022	0.51	0.08
Tenericutes	3.47^a^	3.21^a^	2.53^b^	1.92^b^	2.94	3.28	2.53	2.38	2.78	2.78	0.37	< 0.01	0.15	< 0.01	0.78	0.19
Cyanobacteria	0.08^b^	0.04^b^	0.06^b^	0.32^a^	0.24	0.18	0.05	0.07	0.09	0.12	0.09	< 0.01	0.13	0.50	0.58	0.03
Actinobacteria	0.35^b^	0.37^b^	0.6^ab^	0.87^a^	0.42	0.47	0.49	0.58	0.77	0.55	0.22	< 0.01	0.51	0.12	0.65	0.06
Acidobacteria	0.02^b^	0.01^b^	0.06^b^	0.3^a^	0.02^b^	0.02^b^	0.02^b^	0.06^ab^	0.29^a^	0.16^ab^	0.17	< 0.01	< 0.01	< 0.01	0.017	0.07
Planctomycetes	0.14^b^	0.16^b^	0.17^b^	0.32^a^	0.25	0.14	0.19	0.19	0.18	0.25	0.08	< 0.01	0.31.	< 0.01	0.30	0.33
Verrucomicrobia	0.55	0.65	0.58	0.74	0.47	0.6	0.68	0.76	0.66	0.61	0.09	0.10	0.13	0.73	0.84	< 0.01
Fibrobacteres	0.03^b^	0.05^b^	0.1^ab^	0.17^a^	0.06	0.1	0.13	0.09	0.06	0.06	0.06	< 0.01	0.87	0.030	0.52	0.73
Firmicutes/ Bacteroidetes	8.29^a^	5.35^b^	3.39^ab^	3.31^a^	5.05	5.58	4.95	4.54	5.46	4.93	1.27	< 0.01	0.28	< 0.01	0.83	0.95

Data are expressed as mean ± largest SEM. Sows were regarded as the experimental units, *n* = 6 for each treatment. ^a, b, c^ denote *P <* 0.05. CON, basal corn-soybean diet; DF1, wheat bran-rich diet; DF2, DF3, DF4 and DF5 were wheat bran diet supplemented with 1%, 2%, 3% and 4% of FIBER MIX (guar gum and cellulose, 1:4) at the expense of wheat bran. Linear contrasts (OPC) analysis was used to test the linear and quadratic effects of dietary fiber levels on microbial parameters of sows

The relative abundances of microbiota at genera level (> 1%) were presented in Table 11. The relative abundances of *Clostridium_sensu_stricto_1*, *Methanobrevibacter*, *Ruminococcaceae_NK4A214_group, Turicibacter*, *Terrisporobacter*, *Eubacterium_coprostanoligenes-group, Romboutsia*, *Oscillospira*, *Rikenellaceae_RC9_gut_group*, *Ruminococcaceae_UCG-014*, *Prevotellaceae_NK3B31_group* and *Ruminococcaceae_UCG-010* were affected by stage of gestation (*P <* 0.05 or *P <* 0.01). The relative abundances of *Methanobrevibacter, Treponema_2, Ruminococcaceae_UCG-005, Lachnospiraceae_XPB1014_group, Terrisporobacter, Prevotellaceae_UCG-001, Romboutsia, Prevotellaceae_NK3B31_group* and *Phascolarctobacterium* were affected by dietary fiber levels (*P <* 0.05 or *P <* 0.01). The relative abundances of *Methanobrevibacter*, *Treponema_2 Turicibacter, Rikenellaceae_RC9_gut_group, Prevotellaceae_NK3B31_group, Phascolarctobacterium* and *Ruminococcaceae_UCG-010* were affected by the interaction between gestation stage and diets (*P <* 0.05 or *P <* 0.01).

**Table 11:** The relative abundances of microbiota at the genera level (%, > 1 in at least one sample) in feces of sows during gestation

Item	Gestation stage				Treatment						SEM	*P*-value				
	Day 30	Day 60	Day 90	Day 110	CON	DF1	DF2	DF3	DF4	DF5		Stage	Diets	Stage×Diets	Linear	Quadratic
*Clostridium_sensu_stricto_1*	15.01^a^	14.28^ab^	11.24^bc^	8.73^c^	10.38	12.11	11.78	13.01	13.73	12.86	1.67	< 0.01	0.26	0.21	0.41	0.09
*Streptococcus*	3.89	5.41	5.45	4.45	6.04^ab^	7.05^a^	4.45^abc^	5.38^abc^	3.35^bc^	2.52^c^	1.11	0.08	< 0.01	0.39	< 0.01	0.07
*Methanobrevibacter*	1.06^b^	2.02^a^	2.08^a^	1.86^ab^	2.43	0.69	1.71	1.99	1.61	2.10	0.72	0.02	0.41	< 0.01	0.49	0.58
*Lactobacillus*	2.94	3.21	3.59	3.49	3.36	4.20	4.15	2.76	2.35	3.03	0.92	0.67	0.36	0.37	0.47	0.22
*Ruminococcaceae_NK4A214_group*	5.46^a^	4.45^ab^	3.67^b^	3.54^b^	4.28	4.43	4.05	3.95	4.51	4.48	0.56	< 0.01	0.89	0.30	0.65	0.86
*Christensenellaceae_R-7_group*	1.96	1.81	1.70	1.69	1.77	1.84	1.59	1.96	1.84	1.75	0.23	0.35	0.96	0.20	0.88	0.56
*Ruminococcaceae_UCG-002*	4.81	3.94	3.28	3.36	3.94	4.05	3.60	3.39	4.07	4.05	0.55	0.06	0.88	0.61	0.67	0.65
*Treponema_2*	2.18	2.28	3.15	2.56	3.54^a^	2.25^b^	2.49^b^	2.30^b^	2.23^b^	2.43^b^	0.44	0.07	< 0.01	0.045	0.43	0.03
*Ruminococcaceae_UCG-005*	4.06	3.57	3.41	3.35	3.72	3.80	3.62	3.52	3.36	3.56	0.34	0.14	0.97	0.75	0.73	0.34
*Lachnospiraceae_XPB1014_group*	2.95	3.03	2.82	3.28	2.03^c^	3.05^bc^	3.51^ab^	4.36^a^	2.62^bc^	2.56^bc^	0.55	0.92	< 0.01	0.10	0.31	< 0.01
*Turicibacter*	4.06^a^	3.68^a^	3.13^b^	2.26^c^	3.22	3.00	3.00	3.09	3.97	3.41	0.41	< 0.01	0.21	0.032	0.48	0.25
*Terrisporobacter*	3.44^a^	2.99^a^	2.45^b^	2.24^b^	2.87^ab^	2.52^ab^	2.62^ab^	2.44^b^	3.19^a^	3.04^ab^	0.30	< 0.01	0.021	0.39	0.26	0.86
*Prevotellaceae_UCG-001*	1.29	1.69	1.88	1.45	1.06^b^	0.97^b^	1.29^b^	1.89^ab^	2.58^a^	1.68^ab^	0.56	0.11	0.042	0.14	0.24	< 0.01
*Eubacterium_coprostanoligenes-group*	2.48^a^	2.15^ab^	1.85^ab^	1.55^b^	2.24	2.24	2.06	1.87	1.79	1.86	0.32	< 0.01	0.58	0.083	0.32	0.15
*Romboutsia*	2.00^a^	1.62^ab^	1.40^bc^	1.15^c^	1.66	1.37	1.41	1.31	1.71	1.78	0.19	< 0.01	0.047	0.37	0.13	0.64
*Oscillospira*	1.09^b^	1.03^b^	1.10^b^	1.82^a^	0.86	1.52	1.26	1.61	1.07	1.23	0.31	< 0.01	0.071	0.082	0.95	0.24
*Rikenellaceae_RC9_gut_group*	1.70^b^	2.12^b^	2.59^a^	1.82^b^	2.41	1.98	2.17	1.86	2.03	1.90	0.32	< 0.01	0.25	< 0.01	0.30	0.23
*Ruminococcaceae_UCG-014*	2.17^a^	1.91^ab^	1.77^bc^	1.49^c^	1.96	1.84	1.77	1.53	1.82	2.08	0.15	< 0.01	0.17	0.46	0.12	0.07
*Prevotellaceae_NK3B31_group*	0.53^b^	0.80^b^	1.47^a^	1.38^a^	1.44^a^	0.87^b^	0.96^ab^	1.05^ab^	0.88^b^	1.09^ab^	0.23	< 0.01	0.023	< 0.01	0.91	0.09
*Ruminococcus_1*	1.00	1.13	1.18	1.45	1.19	1.13	1.12	1.22	1.13	1.35	0.21	0.08	0.97	0.51	0.24	0.73
*Phascolarctobacterium*	0.64	0.65	0.65	0.85	0.42^b^	0.88^ab^	1.05^a^	0.76^ab^	0.62^ab^	0.48^b^	0.19	0.37	< 0.01	< 0.01	0.10	0.16
*Ruminococcaceae_UCG-010*	1.21^a^	1.14^a^	0.94^b^	0.82^b^	0.98	1.15	0.99	1.02	1.04	0.98	0.09	< 0.01	0.74	0.047	0.61	0.85

Data are expressed as mean ± largest SEM. Sows were regarded as the experimental units, *n* = 6 for each treatment. ^a, b, c^ denote *P <* 0.05. CON, basal corn-soybean diet; DF1, wheat bran-rich diet; DF2, DF3, DF4 and DF5 were wheat bran diet supplemented with 1%, 2%, 3% and 4% of FIBER MIX at the expense of wheat bran. Linear contrasts (OPC) analysis was used to test the linear and quadratic effects of dietary fiber levels on microbial parameters of sows

### Relationship between dietary fiber, microbiota, SCFAs, serum parameters and sow performance

Pearson’s correlation analysis was performed to investigate the relationships between the microbiota in feces of sows and different fiber components, and Pearson’s correlation coefficients were presented in Table 12. The relative abundances of Proteobacteria in feces of sows were corrected positively dietary CF levels. As shown in Table 11, the relative abundances of Spirochaetes were negatively corrected with levels of NDF, DF and ISF levels (*P <* 0.05), the relative abundances of Cyanobacteria were negatively corrected with levels of NDF, DF, SF and ISF levels (*P <* 0.05), the relative abundances of Acidobacteria were positively corrected with levels of CF and SF levels (*P <* 0.05), and the relative abundances of Verrucomicrobia were positively corrected with levels of CF and SF levels (*P <* 0.05). Relative abundances of Firmicutes, Bacteroidetes, Proteobacteria, Spirochaetes, Tenericutes, Cyanobacteria, Actinobacteria, Acidobacteria, Planctomycetes, Fibrobacteres, Chloroflexi, Synergistetes and ratio of Firmicutes/Bacteroidetes, were significantly corrected with the stage of gestation.

**Table 12:** Bacterial phylum in feces of gestating sows correlated by Pearson’s correlation to different fiber components

Item	CF	NDF	DF	SF	ISF	Gestation stage
Firmicutes	−0.095	− 0.010	− 0.017	− 0.069	− 0.009	− 0.691**
Bacteroidetes	0.034	0.033	0.031	0.036	0.031	0.681**
Euryarchaeota	0.006	−0.153	− 0.141	− 0.049	− 0.153	0.177
Proteobacteria	0.248*	0.139	0.153	0.221	0.141	0.450**
Spirochaetes	−0.190	− 0.266*	− 0.264*	− 0.226	− 0.267*	0.242*
Tenericutes	− 0.117	− 0.089	− 0.090	− 0.113	− 0.086	− 0.576**
Cyanobacteria	− 0.213	− 0.257*	− 0.253*	− 0.238*	− 0.254*	0.363**
Actinobacteria	0.186	0.152	0.157	0.182	0.153	0.458**
Acidobacteria	0.263*	0.154	0.167	0.236*	0.156	0.328**
Planctomycetes	0.025	−0.114	− 0.103	− 0.023	− 0.114	0.369**
Verrucomicrobia	0.207	0.297*	0.291*	0.250*	0.295*	0.227
Fibrobacteres	−0.034	0.083	0.071	0.006	0.081	0.448**
Saccharibacteria	0.028	0.030	0.034	0.031	0.032	−0.056
Chloroflexi	0.223	0.144	0.155	0.206	0.145	0.252*
Synergistetes	0.016	0.139	0.130	0.060	0.139	0.417**
Firmicutes/ Bacteroidetes	−0.021	−0.001	−0.003	− 0.015	0.000	− 0.698**

* denotes *P* < 0.05 and ** denotes *P* < 0.01

As shown in Table 13, levels of CF were corrected with six genera in Firmicutes, three in Bacteroides, one in Euryarchaeota, and two in Spirochaetes in feces of sows (*P <* 0.05 or *P <* 0.01). Levels of NDF were corrected with three genera in Firmicutes, four in Bacteroides, one in Proteobacteria, and one in Spirochaetes in feces of sows (*P <* 0.05 or *P <* 0.01). Levels of DF were corrected with two genera in Firmicutes, four in Bacteroides, one in Proteobacteria, and one in Spirochaetes in feces of sows (*P <* 0.05 or *P <* 0.01). Levels of SF were corrected with one genera in Firmicutes, four in Bacteroides and one in Spirochaetes in feces of sows (*P <* 0.05 or *P <* 0.01). Levels of ISF were corrected with three genera in Firmicutes, four in Bacteroides, one in Proteobacteria and one in Spirochaetes in feces of sows (*P <* 0.05 or *P <* 0.01). Twenty genera in Firmicutes, seven in Bacteroides, two in Proteobacteria, one in Spirochaetes, one in Actinobacteria and one in Fibrobacteres in feces of sows were significantly corrected with gestation stage (*P <* 0.05 or *P <* 0.01).

**Table 13:** Bacterial genera in feces of gestating sows correlated by Pearson’s correlation to different fiber components

Phyla	Genera	CF	NDF	DF	SF	ISF	Stage
Firmicutes	*Clostridium_sensu_stricto_1*	−0.147	0.204	0.208	0.214	0.205	−0.536**
	*Streptococcus*	−0.057	−0.243*	− 0.263*	−0.380**	− 0.244*	0.078
	*Ruminococcaceae_NK4A214_group*	−0.196	0.006	0.010	0.025	0.007	−0.493**
	*Ruminococcaceae_UCG-002*	−0.232	−0.022	− 0.017	−0.001	− 0.020	−0.394**
	*Ruminococcaceae_UCG-005*	−0.227	−0.069	− 0.073	−0.098	− 0.069	−0.269*
	*Ruminococcaceae_UCG-010*	−0.069	0.055	0.050	−0.004	0.056	−0.531**
	*Ruminococcaceae_UCG-014*	0.076	−0.091	−0.080	− 0.020	−0.088	− 0.484**
	*Ruminiclostridium_6*	−0.054	0.213	0.214	0.213	0.212	0.428**
	*Turicibacter*	−0.140	0.052	0.063	0.125	0.053	−0.559**
	*Terrisporobacter*	0.205	−0.010	0.003	0.082	−0.008	−0.494**
	*[Eubacterium]_coprostanoligenes_group*	−0.216	−0.152	− 0.158	−0.194	− 0.152	−0.416**
	*Romboutsia*	0.034	−0.052	−0.036	0.058	−0.050	− 0.531**
	*Oscillospira*	−0.165	0.237*	0.228	0.142	0.236*	0.346**
	*Sarcina*	−0.271*	0.069	0.071	0.101	0.068	0.322**
	*Anaerotruncus*	−0.239*	−0.055	− 0.059	−0.081	− 0.055	−0.242*
	*Blautia*	−0.150	−0.092	− 0.096	−0.092	− 0.094	0.281*
	*Blautia*	−0.150	−0.092	− 0.096	−0.092	− 0.094	0.281*
	*[Anaerorhabdus]_furcosa_group*	−0.259*	0.057	0.039	−0.075	0.055	−0.574**
	*Subdoligranulum*	−0.144	−0.173	− 0.177	−0.193	− 0.174	0.395**
	*Quinella*	−0.149	0.193	0.182	0.119	0.190	0.412**
	*Oscillibacter*	−0.289*	− 0.028	−0.034	− 0.053	−0.029	− 0.429**
	*Lachnospiraceae_XPB1014_group*	−0.199	0.320**	0.303*	0.193	0.316**	0.059
	*Christensenellaceae_R-7_group*	0.278*	0.021	0.024	0.024	0.022	−0.195
	*Phascolarctobacterium*	−0.345**	0.213	0.187	0.045	0.209	0.136
	*Lactobacillus*	−0.105	− 0.042	−0.054	− 0.122	−0.043	0.094
Bacteroidetes	*Prevotellaceae_NK3B31_group*	0.148	−0.258*	−0.250*	− 0.189	−0.258*	0.584**
	*Prevotella_1*	0.168	−0.301*	−0.303*	− 0.278*	−0.302*	0.488**
	*Prevotella_9*	0.058	−0.292*	−0.293*	− 0.273*	−0.293*	0.508**
	*Prevotellaceae_UCG-001*	0.580**	0.240*	0.252*	0.323**	0.240*	0.080
	*Prevotellaceae_UCG-003*	0.056	−0.114	−0.123	−0.167	− 0.115	0.546**
	*Parabacteroides*	0.231	−0.162	−0.149	− 0.065	−0.160	0.489**
	*Alloprevotella*	0.172	−0.007	−0.012	− 0.053	−0.007	0.654**
	*Rikenellaceae_RC9_gut_group*	0.343**	−0.214	−0.217	− 0.203	−0.215	0.160
	*dgA-11_gut_group*	0.332*	−0.058	−0.070	− 0.120	−0.060	0.341**
Euryarchaeota	*Methanobrevibacter*	−0.233*	−0.144	− 0.132	−0.038	− 0.144	0.175
Proteobacteria	*Desulfovibrio*	0.056	0.084	0.097	0.157	0.086	0.441**
	*Succinivibrio*	0.205	−0.319**	−0.307**	−0.227	− 0.317**	0.408**
Spirochaetes	*Treponema_2*	0.644**	−0.333**	−0.329**	− 0.279*	−0.333**	0.185
	*Escherichia-Shigella*	0.239*	−0.010	−0.001	0.057	−0.009	0.411**
Actinobacteria	*Bifidobacterium*	−0.166	0.004	−0.011	−0.084	0.001	0.344**
Fibrobacteres	*Fibrobacter*	0.179	0.078	0.066	0.002	0.076	0.444**

* denotes *P* < 0.05 and ** denotes *P* < 0.01

As shown in Table 14, the relationship between microbiota and fecal SCFAs, serum parameters, and sow performance were presented. The relative abundances of *Lachnospiraceae_XPB1014_group* in feces of sows were positively corrected with serum concentrations of IL-6 and IL-10 and were negatively corrected with the number of total born piglets, number of piglets born alive and birth weight of piglets (*P <* 0.05 or *P <* 0.01). The relative abundances of *Romboutsia* in feces of sows were negatively corrected with serum concentrations of IL-10 (*P <* 0.05). The relative abundances of both *Prevotella_9* and *Prevotella_1* in feces of sows were positively corrected with propionate contents in feces and were positively corrected with serum TNF-α (*P <* 0.05). The relative abundances of *Succinivibrio* were positively corrected with birth weight of piglets (*P <* 0.05).

**Table 14:** Pearson correlations between the gut microbiota, fecal SCFAs, serum parameters and sow performance

	*Streptococcus*	*Treponema_2*	*Lachnospiraceae_XPB1014_group*	*Terrisporobacter*	*Prevotellaceae_UCG*	*Romboutsia*	*Prevotellaceae_NK3B31_group*	*Phascolarctobacterium*	*Prevotella_9*	*Prevotella_1*	*Succinivibrio*
Acetate,	−0.071	0.090	−0.081	−0.001	0.207	0.045	0.167	−0.055	0.180	0.157	−0.003
Propionate	0.052	0.109	−0.079	−0.174	0.156	−0.162	0.199	0.047	0.281*	0.243*	0.079
Butyrate	−0.172	−0.021	− 0.185	0.015	0.128	0.071	0.140	−0.083	0.181	0.087	0.011
Total SCFA	−0.057	0.083	−0.098	−0.041	0.192	−0.001	0.178	−0.036	0.212	0.175	0.019
IL-6	0.009	0.163	0.491**	−0.049	0.138	−0.169	0.025	0.158	0.282	0.242	0.067
IL-10	0.092	−0.003	0.389*	−0.268	0.195	−0.397*	0.074	0.289	0.303	−0.026	0.220
TNF-α	0.072	−0.002	0.130	0.060	−0.157	0.145	−0.145	0.158	−0.425*	−0.413*	− 0.300
IFN-γ	−0.196	0.134	−0.062	− 0.080	0.062	− 0.073	−0.057	− 0.077	−0.118	0.240	0.070
Total born	−0.029	−0.015	− 0.267*	0.067	0.128	0.050	−0.029	−0.015	− 0.162	−0.100	− 0.013
Born alive	−0.166	0.013	−0.270*	0.077	0.182	0.016	0.055	−0.098	−0.071	− 0.033	0.036
Piglet mean BW at birth	−0.026	0.050	−0.215	0.039	0.064	0.032	0.212	−0.121	0.068	0.195	0.302*
Mean litter weight at birth	−0.101	0.035	−0.313**	0.078	0.156	0.038	0.166	−0.129	−0.001	0.102	0.203
Stillborn rate	0.163	−0.023	0.008	−0.016	−0.076	0.036	−0.093	0.106	−0.102	− 0.072	−0.055
Intralitter CV	−0.128	−0.002	0.077	0.025	0.029	0.027	−0.137	−0.188	0.041	−0.066	− 0.157
Duration of farrowing	−0.059	0.015	−0.185	0.008	−0.051	0.041	−0.073	− 0.173	−0.080	− 0.062	0.049

* denotes *P* < 0.05 and ** denotes *P* < 0.01

## Discussion

Due to the various definitions of dietary fiber and different sources and characteristics of dietary fiber between studies, the effects of dietary fiber during gestation on the performance of sows are often inconclusive. One of the confounding factors was that the level of dietary fiber was elevated by adding fiber-rich ingredients (e.g. sugar beet pulp, oat hulls or soybean hulls), which made it hard to distinguish the effects of dietary fiber. To address this concern, the inclusion of dietary fiber was achieved by adding purified dietary fiber guar gum and cellulose in the present study. Guar gum, as a water-soluble fiber, was recently found to change the gut microbiota and the insulin sensitivity for sows [22]. Soluble dietary fiber can be fermented in the small intestine, whereas the microbial metabolism of insoluble dietary fiber (e.g. cellulose) mainly occurred in the hindgut [23]. Therefore, the supplementations of both soluble and insoluble dietary fiber allowed the fermentation of dietary fiber along the total intestinal tract. In the present study, inclusion of guar gum and cellulose resulted in a linear increase of total born piglets and sows receiving the DF5 diets during gestation resulted in 1.78 or 1.49 more piglets born alive compared with the sows in CON or DF1 groups. The greater litter size of sows fed with elevated level of dietary fiber in this study was consistent with previous researches which found that feeding gestating sows the diets high in wheat bran [2], wheat straw (13.35%) [3] or sugar beet pulp (47% NSP) [4] resulted in increases in total number of piglets born or number of piglets born alive. A dose-dependent effect between dietary fiber levels and litter performance was also found by Mroz et al. [24], who found that increasing the inclusion of oat hulls from 0 to 50% in gestation diet linearly increased the litter weight. Other reports, however, reported that the inclusion of high levels of fiber-rich ingredients during gestation did not affect litter size [25–27]. The inconsistency of the effects of dietary fiber inclusion on the reproductive performance remained uncertain but might be linked to differences in the sources or types of dietary fiber. For example, gestation diets supplemented with different ratios of soluble to insoluble fiber, without changing the level of dietary fiber, altered the sow and offspring performance [16, 28]. Sows fed the diet high in soybean hull had lower embryo survival than did the sows fed a diet high in sugar beet pulp [29]. In the present study, no quadratic changes in number of total born piglets or piglets born alive were observed for gestating sows fed different levels of dietary fiber, and the reason might be that purified dietary fiber was supplemented at a low level (from 1% to 4%), which was not high enough to induce a quadratic response.

The current finding revealed that feed intake of sows during lactation was elevated by dietary fiber inclusion during gestation. This agreed with numerous previous studies [4, 6, 30, 31], in which high levels of dietary fiber-rich ingredients in the gestation diet increased feed intake during lactation. Regulation of dietary fiber during gestation on the appetite of sows during lactation can be explained by an increased size of the digestive tract by the physical effects of dietary fiber, which predisposes the sows to consume more feed during lactation. Additionally, a hormonal mechanism was involved in the control of feed intake by dietary fiber, Quesnel et al. [6] reported that greater dietary fiber intake during gestation was able to decrease the hormone leptin in circulation, a critical peptide regulating appetite. However, suckling performance, backfat loss of sows, as well as the weaning-to-estrus interval of post-weaning sows were not affected in this study. An increase in colostrum lipids concentration were found in DF1 and DF2 treatment in the present study, which was in accordance with the results of Loisel et al. [31] who found that colostrum from sows fed with high dietary fiber diet (23.4% DF) contained more lipids than colostrum from sows fed with low dietary fiber diet (13.3% DF) during late gestation. Feyera et al. [32] also found that feeding a high fiber diet for late gestating sows was able to increase the colostrum content by 49%. Changes of lipid content in colostrum in the present study might be caused by the SCFAs produced by dietary fiber. Most of the SCFAs produced by gut fermentation of dietary fiber were absorbed by the intestine [33] and can be taken up by the mammary gland as precursors for de novo synthesis of lipids based on the net mammary uptake fluxes [32]. Colostrum composition experienced dynamic changes with increased suckling of piglets due to a dilution effect with subsequent milk [34]. In the present study, farrowing duration between groups ranged from 234 min in the CON group to 290 min in the DF groups, which might result in differences of timepoints for colostrum collections and thus caused bias of the colostrum content.

Prebiotic fibers appeared to enhance immune function [35], therefore the beneficial effects of dietary fiber on the reproductive performance of sows might be related to alternation of immunity. TNF-α is a biomarker of proinflammatory response whereas IL-10 exerted anti-inflammatory function. Serum TNF-α concentrations were lower and IL-10 levels were elevated for sows fed greater levels of dietary fiber in the present study. This can be partly explained by an increase in microbial metabolites SCFAs, especially an increase in butyrate. SCFAs were enhancers of barrier function in intestinal epithelial cells [36, 37], and butyrate was capable of inhibiting the production of pro-inflammatory cytokine induced by bacterial LPS [36, 38]. Another explanation of the altered immune parameters is that dietary fiber could promote intestinal peristalsis and excretion of the stool to reduce the incidence of gastrointestinal disorder. Constipation, a common gut disorder for gestating sows, may increase the absorption of harmful microbial endotoxins which resulted in a greater risk of infectious diseases [39]. In this study, fecal score, a parameter to reflect the moisture content in feces of sows, was changed by dietary fiber levels and showed that DF diets resulted in alleviation of constipation for gestating sows.

Gut microbiome experienced significant changes during gestation [10, 40]. The observed species, Shannon and Chao1 indexes increased significantly as the progression of pregnancy stage, suggesting that the microbiota diversity was greater at the late gestation stage than in early and mid-term gestation. This finding was in agreement with Zhou et al. [10] and Collado et al. [40] who found increases in Chao 1 index and fecal microbial counts in late gestation. The findings in the current study revealed that gut microbiota experienced a significant change with the progress of gestation at either phylum or genus level. Decrease of the relative abundance of Firmicutes, increase of the relative abundance of Bacteroidetes, as well as decreased Firmicutes/Bacteroidetes ratio with gestation, were associated with increased energy metabolism [41], indicating that gut microbiota of sows at the late gestation possess a greater ability to utilize dietary fiber and promote host energy metabolism. However, the mechanisms responsible for the pregnancy-related increases of microbial diversity remained uncertain, and the meanings of those changes for the gestating sows need our further investigations.

In the present study, correlation analysis revealed significant positive or negative associations between microbiota and sow productive performance, indicating a possible role of microbiota in the control of sow performance by dietary fiber. Gut microbiota varied between sows with high or low stillbirth rate [42], and supplementations of probiotics containing *Bacillus licheniformis* and *Bacillus subtilis spores* [43], *Enterococcus faecium* DSM 7134 [44] or *Bacillus subtilis* C-3102 [45] to gestation diets were observed to enhance the health status and the litter performance of sows. Those results implicated that the gut microbiota could be considered as a target to improve the health status and performance of sows.

## Conclusions

It can be concluded from the present study that the inclusion of purified guar gum and cellulose to increase the dietary fiber level (18.8%) could improve the litter size. The beneficial improvement of reproductive performance for sows was associated with alternations of gut microbiota and immune function.

## Supplementary information

**Additional file 1: Table S1.** Bacterial phyla correlated by Pearson’s correlation to gestation stage and DF level. **Table S2.** Bacterial genera correlated by Pearson’s correlation to gestation stage and DF level.

## Data Availability

All data generated or analyzed during this study are available from the corresponding author on reasonable request.
