# Supplementary material for: Inclusion of purified dietary fiber during gestation improved the reproductive performance of sows
Source: J Anim Sci Biotechnol. 2020 May 12;11:47. doi: 10.1186/s40104-020-00450-5 (PMC7216585; doi:10.1186/s40104-020-00450-5)
Supplement: Supplementary file 1 — Additional file 1: Table S1. Bacterial phyla correlated by Pearson’s correlation to gestation stage and DF level. Table S2. Bacterial genera correlated by Pearson’s correlation to gestation stage and DF level. [file 40104_2020_450_MOESM1_ESM.pdf]

Supplemental Table 1. Bacterial phyla correlated by Pearson's correlation to gestation stage and DF level.

	Stage		DF	
	Coefficient	*P*-value	Coefficient	*P*-value
Firmicutes	-0.691	<0.01	-0.017	0.890
Bacteroidetes	0.681	<0.01	0.031	0.795
Euryarchaeota	0.177	0.137	-0.141	0.236
Proteobacteria	0.450	<0.01	0.153	0.200
Spirochaetes	0.242	0.040	-0.264	0.025
Tenericutes	-0.576	<0.01	-0.090	0.454
Cyanobacteria	0.363	<0.01	-0.253	0.032
Actinobacteria	0.458	<0.01	0.157	0.187
Acidobacteria	0.328	<0.01	0.167	0.161
Planctomycetes	0.369	<0.01	-0.103	0.392
Verrucomicrobia	0.227	0.055	0.291	0.013
Fibrobacteres	0.448	<0.01	0.071	0.554
Saccharibacteria	-0.056	0.638	0.034	0.775
Chloroflexi	0.252	0.033	0.155	0.193
Synergistetes	0.417	<0.01	0.130	0.277
Firmicutes/Bacteroidetes	-0.698	<0.01	-0.003	0.983

DF, total dietary fiber, sum of soluble and insoluble fiber.

Supplemental Table 2. Bacterial genera correlated by Pearson's correlation to gestation stage and DF level.

Phyla	Genera	Stage		DF	
		Coefficient	*P*-value	Coefficient	*P*-value
Firmicutes	*Clostridium_sensu_stricto_1*	-0.536	<0.01	0.208	0.080
	Streptococcus	0.078	0.516	-0.263	0.026
	Ruminococcaceae_NK4A214_group	-0.493	<0.01	0.010	0.935
	Ruminococcaceae_UCG-002	-0.394	<0.01	-0.017	0.885
	Ruminococcaceae_UCG-005	-0.269	0.023	-0.073	0.542
	Ruminococcaceae_UCG-010	-0.531	<0.01	0.050	0.678
	Ruminococcaceae_UCG-014	-0.484	<0.01	-0.080	0.506
	Turicibacter	-0.559	<0.01	0.063	0.601
	Terrisporobacter	-0.494	<0.01	0.003	0.980
	[Eubacterium]_coprostanoligenes_group	-0.416	<0.01	-0.158	0.184
	Romboutsia	-0.531	<0.01	-0.036	0.763
	Oscillospira	0.346	<0.01	0.228	0.055
	Ruminococcus_1	0.326	<0.01	0.025	0.835
	Sarcina	0.322	<0.01	0.025	0.835
	Anaerotruncus	-0.242	0.041	-0.059	0.625
	Blautia	0.281	0.017	-0.096	0.425
	Ruminiclostridium_6	0.428	<0.01	0.214	0.071
	[Anaerorhabdus]_furcosa_group	-0.574	<0.01	0.039	0.745
	Subdoligranulum	0.395	<0.01	-0.177	0.136
	Quinella	0.412	<0.01	0.182	0.126
	Oscillibacter	-0.429	<0.01	-0.034	0.778
Bacteroidetes	*Prevotellaceae_NK3B31_group*	0.584	<0.01	-0.250	0.034
	Prevotellaceae_UCG-001	0.08	0.502	0.252	0.033
	Prevotella_1	0.488	<0.01	-0.303	0.010
	Prevotella_9	0.508	<0.01	-0.293	0.013
	Prevotellaceae_UCG-003	0.546	<0.01	-0.123	0.303
	Parabacteroides	0.489	<0.01	0.008	0.949
	Alloprevotella	0.654	<0.01	-0.012	0.919
Proteobacteria	*Desulfovibrio*	0.441	<0.01	0.097	0.417
	Succinivibrio	0.408	<0.01	-0.307	<0.01
	Escherichia-Shigella	0.411	<0.01	-0.001	0.995
Fibrobacteres	*Fibrobacter*	0.448	<0.01	0.066	0.584
Actinobacteria	*Bifidobacterium*	0.344	<0.01	-0.011	0.926

DF, total dietary fiber, sum of soluble and insoluble fiber.
